# Timing of WIC Enrollment and Responsive Feeding among Low-Income Women in the US

**DOI:** 10.3390/ijerph18147695

**Published:** 2021-07-20

**Authors:** Katelin M. Hudak, Sara E. Benjamin-Neelon

**Affiliations:** 1 Department of Health, Behavior and Society, Johns Hopkins Bloomberg School of Public Health, Johns Hopkins University, Baltimore, MD 21205, USA; 2 Lerner Center for Public Health Promotion, Johns Hopkins Bloomberg School of Public Health, Johns Hopkins University, Baltimore, MD 21205, USA; sara.neelon@jhu.edu

**Keywords:** The Special Supplemental Nutrition Program for Women, infants and children, feeding on demand, infant feeding style

## Abstract

We examined associations between the timing of The Special Supplemental Nutrition Program for Women, Infants, and Children (WIC) enrollment and responsive feeding and assessed food security as a possible effect modifier. We used data from the nationally representative WIC Infant and Toddler Feeding Practices Study-2. Our sample includes women-infant dyads interviewed through the first 13 months of age (*n* = 1672). We dichotomized WIC enrollment as occurring prenatally or after childbirth. The responsive feeding outcome was feeding on demand versus feeding on schedule. We used covariate-adjusted logistic regressions. Of women, 61.8% had a high school education or less and 62.9% lived at 75% or less of the federal poverty guideline. The majority (84.5%) of women enrolled in WIC before childbirth. In unadjusted estimates, 34% of women who enrolled prenatally practiced responsive feeding, compared to 25% of women who enrolled after childbirth. We found no evidence of food security as an effect modifier. In adjusted estimates, women who enrolled in WIC prenatally had 78% higher odds of practicing responsive feeding (OR: 1.78, 95% CI: 1.16, 2.73), compared to women who enrolled after childbirth. Prenatal enrollment in WIC was associated with higher odds of responsive feeding. Future studies should examine how the timing of WIC enrollment relates to responsive feeding in older children and over time.

## 1. Introduction

The Special Supplemental Nutrition Program for Women, Infants, and Children (WIC) is considered a key component of the federal social safety net, providing nutrition education and food benefits for low-income women, infants, and children up to the age of 5 years [1]. There is strong evidence that WIC improves a variety of health outcomes for both mother and child. Women who participate in WIC are more likely to begin prenatal care in the first trimester and are less likely to have a preterm birth [2]. After the 2009 revisions to the WIC food package, pregnant women had healthier overall diets and families consumed more whole grains, low-fat milk, and fruits and vegetables [3,4]. Participation in WIC was associated with healthier infant birth weights [2,5,6,7,8], higher quality diets among preschool-aged children [9], better overall health among children [10], and a lower probability of child food insecurity [11].

Furthermore, the timing of WIC enrollment is also important. Proper maternal nutrition during pregnancy is a key factor in promoting healthy growth and development in utero and can have lifelong consequences [12,13]. Because WIC provides benefits for specific, nutrient-dense foods and nutritional counseling, prenatal WIC enrollment can help low-income women meet their nutritional needs during pregnancy. Several studies have found that earlier participation improves birth outcomes such as infant birth weight [14,15,16].

In addition, WIC has been associated with infant feeding outcomes. The majority of previous studies examining WIC participation and infant feeding focused on breastfeeding, with most studies finding that WIC participation was associated with a lower probability of initiating breastfeeding [2,17,18]. Few studies have assessed other feeding practices, such as responsive feeding. Understanding how WIC participation is associated with responsive feeding is important for multiple reasons. Responsive feeding—feeding infants in response to hunger and satiety cues—is a crucial component in infants learning to self-regulate their intake [19,20,21,22]. Feeding infants in a manner discordant with infant hunger and satiety cues may contribute to rapid weight gain [22,23]. By prompting weight gain in infancy and by hindering infants’ ability to self-regulate, nonresponsive feeding may increase the risk of obesity later in life. WIC endorses responsive feeding and provides resources for WIC staff to communicate with women about identifying and responding to infants’ hunger and satiety cues [24,25]. However, no identified studies have evaluated the effectiveness of WIC’s responsive feeding education on feeding outcomes. Even so, a recent review found that responsive feeding skills, knowledge and understanding of feeding and appetite, and education that supports responsive feeding are key enablers of responsive feeding [26]. In addition, early and repeated exposure to new skills or concepts improve learning outcomes [27,28,29]. Therefore, WIC—and in particular, early enrollment in WIC—has the potential to increase responsive feeding for participating women.

Little is known about what factors influence responsive feeding styles. Maternal race and ethnicity and lower parental education have been associated with a nonresponsive feeding style [30,31,32,33]. Food insecurity—or lacking consistent access to enough food for a healthy life [34]—also has been associated with infant feeding styles [35,36]. Food insecure women were more likely to engage in restrictive or pressuring feeding practices [35], feeding to soothe, and nonresponsive feeding [36]. Women experiencing food insecurity prenatally and during infancy were more likely to exhibit pressuring, indulgent, and laissez-faire feeding styles—styles which ignore the infant’s hunger and satiety cues [36]. The family stress model posits that food insecurity can cause psychological stress for parents, which in turn influences parenting behavior, including feeding style [37,38,39,40]. In addition, there is evidence that food insecurity modifies the connection between nutrition assistance programs and nutritional health outcomes [41]. 

The objective of this study was to examine associations between timing of WIC enrollment and responsive feeding in a national sample of women and infants participating in WIC. We hypothesized that prenatal WIC enrollment would be associated with higher odds of responsive feeding and that the association would be stronger in the presence of food insecurity.

## 2. Materials and Methods

### 2.1. Study Design and Population 

Participants were from the WIC Infant and Toddler Feeding Practices Study−2 (WIC ITFPS-2), a longitudinal study of women and their children 6 years of age and younger. The purpose of WIC ITFPS-2 is to examine feeding practices and nutrition behaviors of WIC-participating women and their children, and how WIC services are associated with feeding and nutrition outcomes [42]. When weighted, WIC ITFPS-2 represents the national population of infants enrolled in WIC in 2013–2014. The WIC Infant and Toddler Feeding Practices Study-2: Infant Year Report comprehensively describes the study design and methods [42]. In brief, women were recruited when they enrolled in WIC, which occurred either prenatally or before their infant was 2.5 months old. Women were eligible to participate if they were at least 16 years old, spoke English or Spanish, and were enrolling in WIC for the first time for that pregnancy or infant. Data were collected via telephone interview, beginning prenatally (if possible), and at 1 and/or 3 months, 5, 7, 9, 11, 13, 15, 18, 24, 30, 36, 42, 48, 54, 60, and 72 months [43]. The main analysis in the dataset included 3777 participants, including a core sample (*n* = 3020) and a supplemental sample (*n* = 757). The supplemental sample was not contacted at each time point, but only at the at the 1- or 3-month interview, and at the 7- and 13-month interviews. The sampling structure combined with survey nonresponse both affect the response rate, which ranged from 61.5% (month 11) to 90.0% (month 1). Supplementary Table S1 shows the response rates by study time point.

We used data from the 1- through 13-month interviews [1] (*n* = 1851 women), and we excluded 179 infants who had long-term medical problems or conditions that may affect what and how the baby eats. Our total sample included 1672 women. The original human subjects’ protections, including informed consent, were overseen by the Westat Institutional Review Board and state health department and local hospital Institutional Review Boards [44]. The Johns Hopkins Bloomberg School of Public Health Institutional Review Board deemed the present analysis of secondary data as exempt.

### 2.2. Exposure: Timing of WIC Enrollment

Our primary exposure was timing of WIC enrollment. Study staff recorded when women enrolled in WIC at baseline, i.e., when women were recruited and enrolled in the study. We operationalized the timing of WIC enrollment as a binary variable to indicate whether the woman enrolled during pregnancy or after childbirth.

### 2.3. Outcome: Responsive Feeding

Our primary outcome was responsive feeding, which we operationalized as a binary variable. Women were asked if they fed their baby according to a schedule or when the baby cries or seems hungry. Potential responses include feeding on schedule, feeding when the baby seems hungry, or both feeding on schedule and when the baby seems hungry. We coded responsive feeding so that nonresponsive feeding (i.e., feeding on schedule or a combination of feeding on schedule and on demand) is equal to zero. We coded feeding on demand (i.e., when the infant cries or seems hungry) as equal to one if the woman reported feeding on demand during any of the interviews [45,46,47]. Responsive feeding was derived from data from the 1-, 3-, 5-, 7-, 9-, 11-, and 13-month interviews. In analyses testing food security as an effect modifier, feeding on demand was derived from data from the 9-, 11-, and 13-month interviews because food security was measured at 7 months.

### 2.4. Other Measures

We identified a priori individual and household factors that have been associated with responsive feeding and WIC participation. Maternal and household variables included age at childbirth (16–19 years, 20–25 years, and 26 years or older), self-reported race (white, Black, or other), ethnicity (Hispanic/non-Hispanic), marital status (married or not married), education level (high school or less, greater than high school), poverty level (% of the poverty guideline), and number of children in the household (continuous), measured at baseline [48,49,50,51,52]. We also controlled for the woman’s work status (full/part time at either the 3, 7, or 13-month interviews, did not work at all) [53,54]. Household food security status was measured during the 7-month interview using the United States Department of Agriculture (USDA) Food Security Survey Module 6-item short form [55]. Food security refers to food security in the past 12 months. We categorized response scores as high/marginal (0–1 affirmative responses), low (2–4 affirmative responses), and very low (5–6 affirmative responses) food security [55].

### 2.5. Analysis

We compared feeding style at each interview (e.g., month 1 and month 3) of women who enrolled prenatally or after childbirth and used Pearson’s chi-squared test to test for significant differences. We computed summary statistics of the outcome, the exposure, and the covariates. We compared the characteristics of women who enrolled prenatally and after childbirth and tested for significant differences with Pearson’s chi-squared (categorical) or two sample t-tests (continuous). We conducted bivariate logistic regression to examine associations between timing of WIC enrollment and responsive feeding. 

To examine effect modification, we tested an interaction between WIC and food security, using a Wald test to assess the interaction. In final models, we conducted multivariable logistic regression, controlling for maternal age at childbirth, maternal race, ethnicity, marital status, maternal education level, household poverty level, and number of children in the household. We checked for the presence of multicollinearity using the variance inflation factor (VIF) [56,57]. We used an alpha level of 0.05 to determine significance. We used the longitudinal (1- or 3-) to 13-month interview weight (which accounts for unequal sampling rates and nonresponse) and Stata 16.1 (StataCorp LP, College Station, TX, USA) for all analyses [58].

## 3. Results

In unadjusted analyses, feeding style was similar between women who enrolled in WIC prenatally or after childbirth (Table 1). Only in month 13 was feeding style significantly different between the two groups, with women who enrolled prenatally using responsive feeding (i.e., feeding on demand) significantly less than those who enrolled after childbirth. Feeding style was largely consistent over time for women who enrolled prenatally, but less so for women who enrolled after childbirth. 

Of women, 24.3% were Black and 59.5% were white (Table 2). Approximately half (49.9%) of women were 26 years or older at childbirth, 39.8% were between the ages of 20–25, and 10.3% were between the ages of 16–19. The majority (61.8%) of women had a high school education or less. Over half (62.9%) lived at 75% or less of the federal poverty guideline. Two-thirds (66.9%) of women reported feeding on schedule, and 33.1% reported feeding their baby on demand. The majority (84.5%) of women enrolled in WIC prenatally. Women who enrolled prenatally were significantly more likely to be Hispanic, to have a high school education or less, and to live at 75% or less of the 2013 poverty guideline. Women who enrolled prenatally were also significantly less likely to be married.

Almost half of all women reported experiencing food insecurity; 30.5% had low food security and 16.6% had very low food security. Women who enrolled in WIC prenatally had significantly higher food security (X^2^ (1, *n* = 1672) = 6.44, *p* = 0.04). However, when we tested for effect modification, we found no evidence of an interaction between timing of WIC enrollment and food security (*p* = 0.51) [2]. Therefore, we fit a main effect model only. When we assessed our final model for multicollinearity, the VIFs ranged from 1.03 to 3.12, indicating that multicollinearity was not an issue [56,57]. In multivariate logistic regression, the odds of responsive feeding for women who enrolled in WIC prenatally were approximately 78% (odds ratio [OR]: 1.78, 95% confidence interval [CI]: 1.16, 2.73) higher than for women who enrolled after childbirth (Table 3). 

## 4. Discussion

In a large, nationally representative study of WIC participants, we found that women who enrolled in WIC prenatally had greater odds of engaging in responsive feeding practices during infants’ first year of life. However, we did not observe any evidence that food insecurity modified this association.

Our study supports previous findings that WIC is associated with infant feeding [17,59,60,61]. However, few studies are directly comparable; the majority of previous studies focused on the relation between WIC participation and breastfeeding initiation and duration. Several studies found that compared to non-participating women, WIC participants were less likely to initiate breastfeeding or to breastfeed exclusively [2,17,18].

Other studies examined the timing of WIC enrollment. Ziol-Guest and Hernandez [61] found that compared to women who enrolled in WIC after childbirth, women who enrolled during their first or second trimester were less likely to initiate breastfeeding. In contrast, Schwartz, et al. [62] found that when combined with breastfeeding advice, prenatal WIC enrollment increased breastfeeding initiation. However, ours is the first study identified to explicitly examine the association between the timing of WIC enrollment and responsive feeding.

Feeding practices in early life, like responsive feeding, shape healthy emotional and cognitive development, as well as infant weight outcomes and dietary preferences [22,63]. Recent literature reviews conducted by the Robert Wood Johnson Foundation and Healthy Eating Research identified responsive feeding as a key factor for promoting healthy weight in infants and toddlers [63]. Protecting against obesity early is crucial; childhood obesity has tripled in the past four decades [64], and trajectories of high weight can begin as early as infancy [65]. In 2014, 12.3% of low-income infants ages 3–23 months were above the 95^th^ percentile for age- and sex-specific weight-for-length [66]. Promoting responsive feeding through WIC is one potential avenue by which to mitigate the risk of obesity in low-income infants and young children [22,67,68,69,70,71]. Furthermore, responsive interactions between parents and young children promote the development of a secure attachment style, which is important for emotional and social development [72,73]. In addition, responsive play and interactions have been associated with more sophisticated cognitive and language abilities during the second year of life [72,74,75].

The timing of WIC enrollment may operate via a window of receptivity to influence a woman’s feeding style. Women and adults in general are more receptive to information when they are in a calm environment and have had sufficient sleep [76,77]. Adults are less likely to respond to information when their mental energy is depleted, when they are sleep-deprived, and when they are under a time pressure [76,77]. In a busy, stressful environment that involves multi-tasking, such as daily life with a newborn, the hectic setting may tax processing resources, leading to lower receptivity to information [76,77]. Many women report experiencing increased stress, anxiety, lack of sleep, and time constraints in the months following childbirth [78,79,80,81]. Women with low socioeconomic status—such as women participating in WIC—are more likely to experience anxiety in the postpartum period [82,83]. Therefore, there may be a window of receptivity before the baby is born during which time women are more open to information. Learning information about responsive feeding practices from WIC staff before the baby is born may make the woman more receptive to the information.

In our study, we did not find evidence of an interaction between timing of WIC enrollment and food security. Thus, our findings suggest that the effect of timing of WIC enrollment did not vary by food security status, which was contrary to our hypothesis. Our hypothesis was based on previous studies in which authors found food insecurity to be associated with less responsive feeding styles [35,36]. Even though we did not observe an interaction between WIC and food security, other research suggests that the positive effects of WIC tend to be concentrated among the most disadvantaged subgroups of women and children, e.g., lowest-income families, women who did not finish high school, teen mothers, and Black and Hispanic infants [2,6,9,16]. 

This study has limitations. First, the sample for this study includes only WIC participating women and, therefore, may not be generalizable to the population. Second, it would be ideal to have an outcome variable that better captures the nuances of a woman’s feeding behavior, e.g., the percentage of time that a woman uses responsive feeding practices compared to nonresponsive feeding practices. Because a portion of study participants did not participate in all of the interviews, either because of survey nonresponse or because of sample design, our ability to create a such a variable was limited. Therefore, we chose to simplify and use a binary outcome. Even so, this coding mirrors other studies on responsive feeding [45,47]. Furthermore, our outcome variable, responsive feeding, is based on self-reported survey data. In some instances, participants may respond in ways that they deem to be more socially desirable [84]. If respondents in the WIC ITFPS-2 study did not answer specific questions accurately, e.g., reporting higher levels of responsive feeding, this could potentially result in social desirability bias.

Fourth, our study was cross-sectional, and we were not able to examine the connection between the timing of WIC enrollment and responsive feeding over time. To clarify, because women were recruited and enrolled in the study when they enrolled in WIC for the first time for that pregnancy/infant, we were unable to look at feeding style before women began participating in WIC. Next, we measured food security using the USDA 6-item short form, which precludes us from distinguishing between marginal and high food security [55]. There is evidence that marginal food security can have negative implications for child health [85]. Therefore, our inability to separate out marginal food security may have partially hindered us from identifying food security as a significant effect modifier. 

Finally, our finding that women who enrolled in WIC prenatally were more likely to practice responsive feeding may be in part due to selection. Women who enroll in WIC earlier may be systematically different than those who enroll after childbirth. For example, they may be more motivated to do everything possible to have a healthy pregnancy and a healthy baby. If this is the case, then the significant association we see between early WIC enrollment and responsive feeding may be due to underlying factors that drive both early WIC enrollment and responsive feeding. Although women who enroll in WIC prenatally are similar on observable characteristics on some dimensions (Table 2), we cannot rule out the possibility that sample selection is part of the story. 

Future studies should utilize a design that mitigates selection bias when examining the relation between WIC enrollment and infant feeding style. Furthermore, future studies should examine whether there are sustained associations between timing of WIC enrollment and responsive feeding and investigate the mechanism(s) through which WIC influences responsive feeding. Finally, additional research should investigate the relation between the timing of WIC enrollment and infant feeding style in older children and over time. 

## 5. Conclusions

We found that prenatal WIC enrollment increased the odds of women using a responsive feeding style, compared to women who enrolled after childbirth. Given that the low-income, WIC-eligible population faces a higher risk of childhood obesity, the association between early WIC enrollment and responsive feeding is especially important. Responsive feeding during infancy may play a key role in the development of self-regulation and in protecting against child obesity. WIC and public health educators should continue to promote responsive feeding practices with women during pregnancy. Furthermore, nutrition educators should place additional emphasis on responsive feeding during WIC counseling sessions in infants’ first year of life to reinforce the importance of feeding in response to hunger and satiety cues. 

## Figures and Tables

**Table 1:** Responsive feeding over time in WIC ITFPS-2.

	Percentage (*n*)		
	Prenatal	Postnatal	*p*-Value ^a^
Feeding Style	*n* = 1476 ^b^	*n* = 196 ^b^	
Month 1			0.27
Schedule	56.2% (790)	51.4% (74)	
On demand	43.8% (616)	48.6% (70)	
Month 3			0.90
Schedule	60.3% (878)	60.8% (118)	
On demand	39.7% (577)	39.2% (76)	
Month 5			0.10
Schedule	58.3% (860)	52.0% (102)	
On demand	41.7% (615)	48.0% (94)	
Month 7			0.51
Schedule	58.4% (847)	60.8% (118)	
On demand	41.6% (604)	39.2% (76)	
Month 9			0.44
Schedule	56.5% (814)	53.6% (104)	
On demand	43.5% (626)	46.4% (90)	
Month 11			0.19
Schedule	57.8% (817)	52.7% (97)	
On demand	42.2% (597)	47.3% (87)	
Month 13			0.04
Schedule	62.9% (332)	49.2% (30)	
On demand	37.1% (196)	50.8% (31)	

^a^ *p*-value for the Pearson’s chi-squared test. ^b^ Differences in sample numbers for a given timepoint may not add up to the total sample size of the group because not all women participated in each interview. For example, 90% of the women that enrolled in WIC ITFPS-2 completed the month 1 interview, whereas 74% completed the month 13 interview. In addition, women who enrolled after childbirth may not have completed the month 1 interview, depending on how old their infant was when they enrolled. WIC: Special Supplemental Nutrition Program for Women, Infants, and Children; WIC ITFPS-2: WIC Infant and Toddler Feeding Practices Study-2.

**Table 2:** Characteristics of women and infants in the WIC ITFPS-2 study: Infant year (*n* = 1672).

	Percentage (*n*)			
	Total	Prenatal	Postnatal	*p*-Value ^a^
Characteristics	*n* = 1672	*n* = 1476	*n* = 196	
Age at childbirth				0.31
16–19 years	10.3% (173)	10.6% (157)	8.2% (16)	
20–25 years	39.8% (665)	40.1% (592)	37.2% (73)	
26 years or older	49.9% (834)	49.3% (727)	54.6% (107)	
Race				0.35
White	59.5% (995)	58.9% (869)	64.3% (126)	
Black	24.3% (407)	24.7% (365)	21.4% (42)	
All other	16.0% (285)	16.4% (242)	14.3% (28)	
Ethnicity				0.01
Not Hispanic	59.0% (987)	57.8% (853)	68.4% (134)	
Hispanic	41.0% (685)	42.2% (623)	31.6% (62)	
Married ^b^	33.7% (564)	31.9% (471)	47.4% (93)	<0.001
Education				0.04
High school or less	61.8% (1030)	62.7% (922)	55.1% (108)	
More than high school	38.2% (637)	37.3% (549)	44.9% (88)	
Number of children in household ^c^ (mean [SD])	2.3 (1.2)	2.3 (1.2)	2.4 (1.2)	0.41
Poverty guideline 2013				0.01
75% or less	62.9% (1152)	63.8% (941)	56.6% (111)	
Above 75% & less than 130%	27.2% (455)	27.2% (402)	27.0% (53)	
Above 130%	9.9% (165)	9.0% (133)	16.3% (32)	
Work status				0.78
No work	46.8% (783)	47.2% (697)	43.9% (86)	
Full/part time	53.2% (889)	52.8% (779)	56.1% (110)	
Timing of WIC enrollment				
Prenatal	84.5% (2923)	-	-	
Postnatal	15.5% (538)	-	-	
Responsive feeding: 1–13 months				0.01
Feeding on schedule	66.9% (1119)	65.9% (972)	75.0% (147)	
Feeding on demand	33.1% (553)	34.1% (504)	25.0% (49)	
Responsive feeding: 9–13 months				0.18
Feeding on schedule	60.2% (1002)	59.6% (876)	64.6% (126)	
Feeding on demand	39.8% (662)	40.4% (593)	35.4% (69)	
Food security ^d^				0.04
High or Marginal	52.9% (884)	53.5% (789)	48.5% (95)	
Low	30.5% (510)	30.8% (454)	28.6% (56)	
Very low	16.6% (278)	15.8% (233)	23.0% (45)	

^a^*p*-value for the Pearson’s chi-squared (categorical) or two sample t-test (continuous) comparing women who enrolled in WIC prenatally and those who enrolled after childbirth. ^b^ Not married includes divorced and widowed. ^c^ Number of children living in the household refers to baseline (either the prenatal, 1 month, or 3 month interview). ^d^ Food security refers to food security in the past 12 months and is calculated from the U.S. Department of Agriculture Food Security Survey Module 6-item short form [55]. SD: standard deviation; WIC: Special Supplemental Nutrition Program for Women, Infants, and Children; WIC ITFPS-2: WIC Infant and Toddler Feeding Practices Study-2.

**Table 3:** Timing of WIC enrollment and adjusted odds ratios (95% confidence intervals) of responsive feeding in WIC ITFPS-2 ^a^.

	Responsive Feeding OR (95% CI)
	(*n* = 1667)
Prenatal WIC enrollment ^b^	1.78 **
	[1.16, 2.73]
Age at childbirth	
16–19 years	Ref.
20–25 years	0.74
	[0.47, 1.17]
26 years or older	1.01
	[0.64, 1.57]
Race	
White	Ref.
Black	0.90
	[0.62, 1.28]
All other	1.33
	[0.95, 1.85]
Hispanic	1.36 * [1.03, 1.79]
Married	1.37 *
	[1.03, 1.83]
Education	
<High school	Ref.
>High school	0.91
	[0.69, 1.20]
% of poverty guideline	
75% or less	Ref.
75%–130%	1.07
	[0.80, 1.44]
Above 130%	1.03 [0.65, 1.62]
Number of children in household	0.97 [0.87, 1.09]
Work status	
No work	Ref.
Full/part time work	1.40 * [1.08, 1.81]

95% confidence intervals in brackets * *p* < 0.05, ** *p* < 0.01. ^a^ Responsive feeding was coded as equal to 0 (i.e., nonresponsive feeding) if the woman reported feeding the infant on schedule or both on schedule and on demand during any of the included interviews. Responsive feeding was coded as equal to 1 if the woman reported feeding on demand (i.e., when the infant cries or seems hungry). Weights were used in all analyses (WCM1_3_13LCOR). Infants who had feeding abnormalities as reported in any interview were excluded. ^b^ Timing of WIC enrollment was recorded at baseline when women enrolled in WIC for the first time for their current pregnancy. Feeding on demand was derived from data from the 1-, 3-, 5-, 7-, 9-, 11-, and 13-month interviews. The model controlled for maternal age at childbirth, maternal race and ethnicity, marital status, maternal education level, household poverty level, and number of children in the household. OR: odds ratio; WIC: The Special Supplemental Nutrition Program for Women, Infants, and Children; WIC ITFPS-2: WIC Infant and Toddler Feeding Practices Study-2.

## Data Availability

For this paper, we analyzed data from the infant year of the WIC Infant and Toddler Feeding Practices Study-2. See https://www.fns.usda.gov/wic/wic-infant-and-toddler-feeding-practices-study-2-infant-year-report (accessed on 8 May 2021).

## Supplementary Materials

The following are available online at https://www.mdpi.com/article/10.3390/ijerph18147695/s1, Table S1: Response rates of the analysis sample by study time point (*n* = 3777) and Table S2: Timing of WIC enrollment and odds ratios (95% confidence intervals) of responsive feeding in WIC ITFPS-2^a^.
