# Supplementary material for: Timing of WIC Enrollment and Responsive Feeding among Low-Income Women in the US
Source: Int J Environ Res Public Health. 2021 Jul 20;18(14):7695. doi: 10.3390/ijerph18147695 (PMC8305462; doi:10.3390/ijerph18147695)
Supplement: Supplementary file 1 [file ijerph-18-07695-s001.zip › ijerph-1247624-supplementary.pdf]

International Journal of
Environmental Research and Public Health

MDPI

Table S1. Response rates of the analysis sample by study time point (n = 3777).

Interview	% (n)
Prenatal	70.1 (2,649)
Month 1	90.0 (3,398)
Month 3	76.3 (2,881)
Month 5	69.8 (2,636)
Month 7	83.0 (3,134)
Month 9	64.9 (2,451)
Month 11	61.5 (2,322)
Month 13	74.3 (2,807)

Table S2. Timing of WIC enrollment and odds ratios (95% confidence intervals) of responsive feeding in WIC ITFPS-2[a].

	Responsive feeding OR
	(n = 1667)
Prenatal WIC enrollment [b]	1.81 ** [1.18, 2.79]
Food security	
High or marginal	Ref.
Low	1.01 [0.76, 1.33]
Very low	1.26 [0.88, 1.81]
Age at childbirth	
16–19 years	Ref.
20–25 years	0.73 [0.47, 1.15]
26 years or older	0.99 [0.63, 1.54]
Race	
White	Ref.
Black	0.90 [0.63, 1.29]
All other	1.34 [0.96, 1.86]
Hispanic	1.37* [1.05, 1.81]
Married	1.40* [1.04, 1.86]
Education	
< High school	Ref.

> High school	0.90 [0.68, 1.18]
% of poverty guideline	
75% or less	Ref.
75% - 130%	1.08 [0.81, 1.44]
Above 130%	1.04 [0.65, 1.65]
Number of children in household	0.97 [0.87, 1.09]
Work status	
No work	Ref.
Full/part time work	1.40* [1.08, 1.81]

95% confidence intervals in brackets * $p < 0.05$, ** $p < 0.01$. [a] Responsive feeding was coded as equal to 0 (i.e. nonresponsive feeding) if the woman reported feeding the infant on schedule or both on schedule and on demand during any of the included interviews. Responsive feeding was coded as equal to 1 if the woman reported feeding on demand (i.e. when the infant cries or seems hungry). Weights were used in all analyses (WCM1_3_13LCOR). Infants who had feeding abnormalities as reported in any interview were excluded.[b] Timing of WIC enrollment was recorded at baseline when women enrolled in WIC for the first time for their current pregnancy. Feeding on demand was derived from data from the 9 -, 11-, and 13 – month interviews. OR: odds ratio; WIC: The Special Supplemental Nutrition Program for Women, Infants, and Children.
